# Network, Transcriptomic and Genomic Features Differentiate Genes Relevant for Drug Response

**DOI:** 10.3389/fgene.2018.00412

**Published:** 2018-09-25

**Authors:** Janet Piñero, Abel Gonzalez-Perez, Emre Guney, Joaquim Aguirre-Plans, Ferran Sanz, Baldo Oliva, Laura I. Furlong

**Affiliations:** ^1^Integrative Biomedical Informatics Group, Research Programme on Biomedical Informatics, Hospital del Mar Medical Research Institute, Department of Experimental and Health Sciences, Universitat Pompeu Fabra, Barcelona, Spain; ^2^Institute for Research in Biomedicine, The Barcelona Institute of Science and Technology, Barcelona, Spain; ^3^Structural Bioinformatics Group, Research Programme on Biomedical Informatics, Department of Experimental and Health Sciences, Universitat Pompeu Fabra, Barcelona, Spain

**Keywords:** drug response, pharmacogenomics, adverse drug reaction, genomics, network biology, gene expression

## Abstract

Understanding the mechanisms underlying drug therapeutic action and toxicity is crucial for the prevention and management of drug adverse reactions, and paves the way for a more efficient and rational drug design. The characterization of drug targets, drug metabolism proteins, and proteins associated to side effects according to their expression patterns, their tolerance to genomic variation and their role in cellular networks, is a necessary step in this direction. In this contribution, we hypothesize that different classes of proteins involved in the therapeutic effect of drugs and in their adverse effects have distinctive transcriptomics, genomics and network features. We explored the properties of these proteins within global and organ-specific interactomes, using multi-scale network features, evaluated their gene expression profiles in different organs and tissues, and assessed their tolerance to loss-of-function variants leveraging data from 60K subjects. We found that drug targets that mediate side effects are more central in cellular networks, more intolerant to loss-of-function variation, and show a wider breadth of tissue expression than targets not mediating side effects. In contrast, drug metabolizing enzymes and transporters are less central in the interactome, more tolerant to deleterious variants, and are more constrained in their tissue expression pattern. Our findings highlight distinctive features of proteins related to drug action, which could be applied to prioritize drugs with fewer probabilities of causing side effects.

## Introduction

Drugs exert their effect acting at different scales of biological organization. At the cellular level, the effect of a drug is the result of its interaction with the target(s), which in time may lead to a variety of cellular responses, such as the alteration of the expression of a set of genes, changes in intracellular signaling pathways, or changes in the localization of proteins, that result in specific cell phenotypic responses. At the organism level, drug absorption, distribution, metabolism, and excretion (ADME) also contribute to modulate the response to the drug. Nevertheless, our understanding of the molecular events elicited by drugs, which result on their therapeutic effects or adverse reactions, is still very limited.

The response to drug treatment is also influenced by the genetic background of an individual (Madian et al., 2012). Nowadays, for some drugs, the impact of genetic variability is well established. More than 200 FDA approved drugs include pharmacogenomic labeling (US Food and Drug Administration ^1^), and pharmacogenomic screenings for known biomarkers are routinely carried out in large hospitals (Roden et al., 2011; van der Wouden et al., 2017; Weinshilboum and Wang, 2017). In particular, the genomic variation of genes involved in drug metabolism and its impact on drug response has been extensively studied (Shenfield, 2004; Pinto and Dolan, 2011; Kozyra et al., 2017) (for recent reviews see Ahmed et al., 2016; Lauschke et al., 2018), Nevertheless, only few studies have probed the role of the genomic variability of drug targets. The results of these studies imply that there is a high frequency of variants impacting protein function in drug targets (Schärfe et al., 2017), pharmacogenes (Wright et al., 2018) and GPCRs (Hauser et al., 2018) in the population. In spite of these studies, we still lack a detailed characterization of the genomic variation of the full spectrum of genes relevant for drug response, including drug targets, ADME genes and genes associated to the side effects of drugs, and their impact on drug response phenotypes.

In the field of systems pharmacology, the study of the perturbations elicited by drugs within the context of cellular networks has provided insight into the molecular mechanisms leading to drug action, including their adverse reactions (Berger and Iyengar, 2011). Network analysis of omics data has been used to identify modules associated with drug response and toxicity (Berger et al., 2010; Bauer-Mehren et al., 2012), to characterize the therapeutic (Yildirim et al., 2007; Guney et al., 2016) and adverse effect of drugs (Guney, 2017), and to explain the similarity of side effects of different drugs (Brouwers et al., 2011).

**FIGURE 1:**
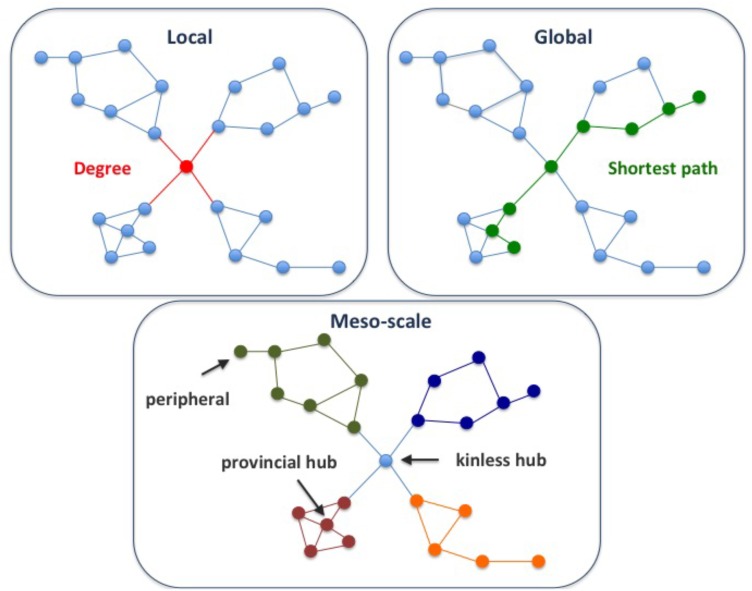
Multi-scale network properties and cartographic roles. The multi-scale network analysis involves exploring the network properties of the proteins at different scales, namely local, meso-scale, and global. The degree of a node is a *local* network property, since it considers the first direct neighbors of a node, while the shortest path is a *global* network property, since we need to count paths between pairs of nodes across the whole network. Finally, the *meso-scale* network properties represent the organization of the network into clusters or modules. The meso-scale connectivity features of each protein can be characterized with the cartographic role classification scheme proposed by Guimerà and Amaral, 2005, namely ultra-peripheral, peripheral, non-hub connector, non-hub kinless, provincial hubs, connector hubs and kinless hubs (see **Supplementary Figure S1** for more information). Thus, focusing on how individual nodes are positioned in the modular (meso-scale) structure of the network, we can identify proteins that play different functions, such as mainly connected to other proteins within their modules (e.g., provincial hub), and those proteins that serve as bridges between modules (e.g., kinless hub).

A key goal of network analysis is to connect network structure to function. For example, multi-scale network analysis allowed distinction of different classes of disease genes based on their connectivity patterns in the human protein-protein interaction network, or interactome (Berenstein et al., 2015; Piñero et al., 2016a). The multi-scale network analysis involves the exploration of the network properties of the proteins at *local*, *meso* and *global* scales. *Local* properties of a protein in a network pertain to its direct interactions with other nodes (**Figure 1**). Examples of local properties are the degree of a node (the number of direct neighbors), or the clustering coefficient (the density of links in the node’s immediate neighborhood). *Global* properties consider the links across the whole network. An example is the betweenness centrality (the proportion of shortest paths passing through a node in a network). Finally, the *meso-scale* network properties are related to the organization of the network into clusters or modules, that represent functional units in the cell (Hartwell et al., 1999). Exploring the connectivity of proteins at the meso-scale level can shed light on the modular organization of the interactome, potentially revealing the regulation of cellular processes.

Here we provide a comprehensive characterization of genomic, transcriptomic and network topological features of genes relevant to drug response. We carefully selected three sets of proteins relevant to pharmacokinetics and pharmacodynamics: drug targets, proteins associated to phenotypes of drug toxicity, and proteins involved in the transport and metabolism of drugs. By leveraging on data from large scale genomic and transcriptomic initiatives and the reconstructions of the human protein interactome, we characterized the tolerance to deleterious genomic variability across human populations, the multi-scale network properties, and the expression across human tissues of proteins involved in the therapeutic and toxic response to drugs.

## Materials and Methods

### The Data

#### Drug Targets (TARGET)

**FIGURE 2:**
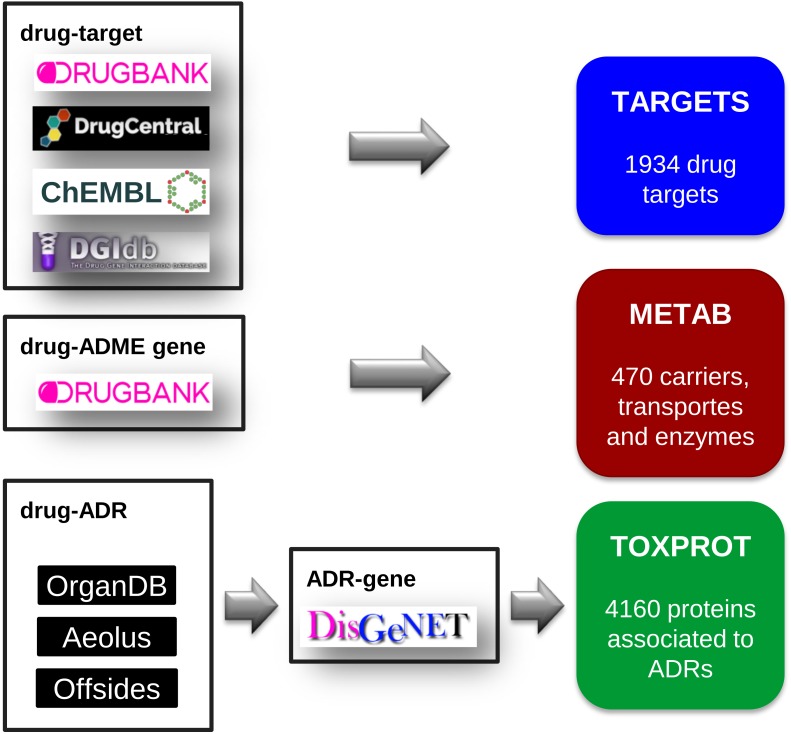
The assembly of the three sets of proteins relevant for drug response: the drug target set (TARGET), the proteins involved in drug transport and metabolism (METAB), and the proteins associated to side effects (TOXPROT).

We compiled a comprehensive set of drug target proteins (referred as TARGET hereafter) that mediates the therapeutic effects of the drugs by integrating data from several repositories: DrugBank, version 5.0.7 (Wishart et al., 2018), DrugCentral, data downloaded on September, 2017 (Ursu et al., 2017), DGIdb, version 3.0 (Cotto et al., 2017), and ChEMBL, version 23 (Bento et al., 2014). We then mapped all the drugs to DrugBank identifiers, and all proteins to NCBI Gene identifiers. From DrugBank, we included only targets for approved or investigational drugs. From DrugCentral, we kept only targets in the Tclin category. From DGIdb we considered drug-target associations from “ChEMBL,” “GuideToPharmacology,” “Tdg Clinical Trial,” “FDA,” “TEND,” and “TTD.” From ChEMBL, we kept the drug-target relationships for which we could find a corresponding DrugBank identifier. Finally, we removed any protein present in the METAB set (see below). The TARGET set was composed of 1,934 proteins, targeting 2,829 drugs (**Figure 2**).

#### Drug Carriers, Transporters and Metabolism Enzymes (METAB)

We retrieved the proteins that act as drug transporters, drug carriers, and enzymes involved in drug absorption, distribution, and metabolism from DrugBank. We mapped all proteins to NCBI Gene identifiers. We thus obtained the METAB set, composed of 470 proteins involved in the transport and metabolism of 1,519 drugs (**Figure 2**).

#### Proteins Associated to Drug Toxicity (TOXPROT)

We assembled a set of proteins associated to the side effects or toxicity phenotypes of the drugs included in this study. To do this, we first collected drug side effects, and drug therapeutic indications. The therapeutic indications were obtained from SIDER, version 4.1 (Kuhn et al., 2016), AEOLUS (Shah, 2016), CTD, revision 15142 (Davis et al., 2017), repoDB, version 1.2 (Brown and Patel, 2017), and ChEMBL, version 23. We mapped drugs to DrugBank identifiers, and disease identifiers to the Unified Medical Language System (UMLS, version 2016AB) Concept Unique Identifiers (CUIs) (Bodenreider, 2004). We only kept therapeutic indications reported by more than one source. The data of Adverse Drug Reaction (ADRs) was retrieved from 3 sources: Offsides (Tatonetti et al., 2012), AEOLUS, and ORGANDB (Mannil et al., 2015) (all files were downloaded on September, 2017). As we did with drug therapeutic indication data, we used UMLS CUIs to harmonize phenotypes and DrugBank identifiers to represent drugs.

Next, we filtered out phenotypes annotated to the UMLS semantic types “Patient or Disabled Group,” “Professional or Occupational Group,” “Therapeutic or Preventive Procedure,” “Medical Device.” To produce a high confidence dataset, we only kept associations reported by the three sources (Offsides, AEOLUS, and ORGANDB). From this set of drug-ADRs we removed the phenotypes/diseases that overlapped with the therapeutic indications of drugs. This produced a list of 12,213 drug-side effects pairs involving 593 drugs and 718 side effects. Finally, we used DisGeNET Curated (version 5.0) (Piñero et al., 2016b) to obtain a list of 4,160 genes associated to 452 ADRs, which we refer as TOXPROT throughout the text (**Figure 2**).

**FIGURE 3:**
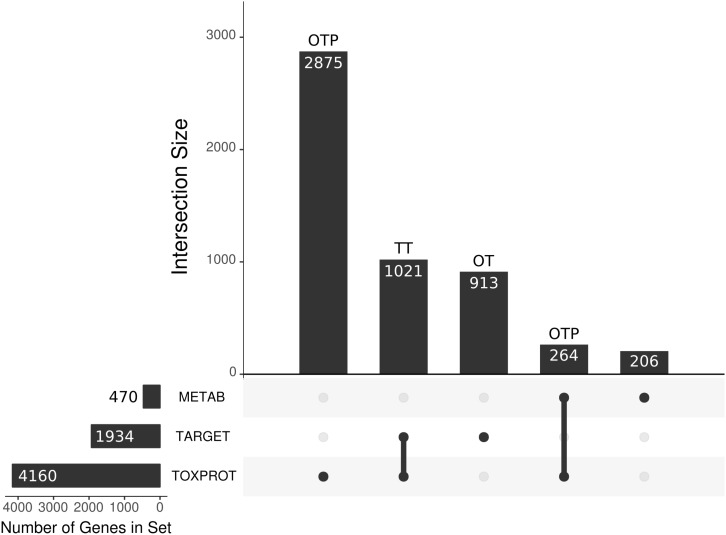
We present the main sets of proteins under study, and their overlaps. The horizontal bars on the left correspond to the three larger categories: the METAB set, constituted of 470 proteins that are involved in drug absorption, distribution, metabolism, and excretion; the drug target set (TARGET), composed of 1,930 proteins; and the set of 4,160 proteins associated to side effects (TOXPROT); the intersections among these categories are represented with the filled dots in the matrix. The TARGET set shares 1,021 proteins with the TOXPROT set and we refer to those drug targets associated toxicity as the TT set. The OT (only targets) set are the TARGET proteins that are not included in the TOXPROT set (913 proteins). The only TOXPROT proteins (OTP) set is composed by 3,139 TOXPROT proteins that are not in the TARGET set. In the figure, it corresponds to the two bars marked as OTP.

Due to the overlap between the TARGET and TOXPROT sets of proteins (see **Figure 3**) we separately assessed the properties of the overlapping subset of genes (TT), the genes annotated uniquely as drug targets (OT), and those annotated only as associated to drugs toxicity (OTP).

#### TARGET, TOXPROT, and METAB Protein Classes

We used data from Pharos, version 4.6.2 (Nguyen et al., 2017) to classify the drug targets in seven categories: GPCR, Transcription Factor, Enzyme, Kinase, Transporter, Ion Channel, and Nuclear Receptor (NHR). We extended this classification to the TOXPROT using the equivalent terms from the classification from Panther database, version 13.0 (Mi et al., 2017) in the file^2^. For METAB, we used the classification provided by DrugBank: transporters, carriers, enzymes.

### The Network Analysis

#### Protein Interaction Data

We built two high-confidence protein-protein interaction networks (PIN) using data from INBIOMAP (Li et al., 2017) and from HIPPIE (Alanis-Lobato et al., 2017), two resources that integrate information from several other sources, and provide a reliability score that allows to filter the interactions. To build the INBIOMAP network, we downloaded the file^3^ (version 2016_05_31). We removed predicted interactions, and kept only interactions with score greater than 0.15. In the case of the HIPPIE-based network, we downloaded the file ^4^ (version 2.1). To produce a high confidence network, we filtered out all interactions with score smaller than or equal to 0.7 (keeping ∼25% of HIPPIE). From both PINs, to obtain a biologically meaningful modular representation of the network, we removed genes with degree higher than 300, such as chaperones and ubiquitins.

We also compiled 4 organ-specific interactomes using GTEx data (version 7.0) for brain, liver, kidney and heart. Briefly, we first mapped the ENSEMBL gene identifiers in the GTEx expression matrix to NCBI Gene identifiers. In the cases of brain, and heart, we merged the gene expression of different zones, and computed the median value of expression for each gene (Melé et al., 2015). Then, we removed from the PINs all interactions involving at least one gene with TPM < 1 in the corresponding tissue.

#### Network Cartographic Roles

To assign cartographic roles in the PINs to each protein, we computed the z (within-module degree) and P (participation coefficient) of each gene following the protocol described in Piñero et al. (2016a). Briefly, we clustered the PINs using the Infomap algorithm (Rosvall and Bergstrom, 2008) and calculated *z* and *P* using equations (1) and (2), respectively.

(1) $$Z_{i}=\frac{k_{i}-{\bar{k}}_{\mathrm{ci}}}{\sigma {\bar{k}}_{\mathrm{ci}}}$$

where k_i_ is the number of links of node i to other nodes in its module, ${\bar{k}}_{\mathrm{ic}}$ is the mean degree of all nodes in cluster c_i_, and σ ${\bar{k}}_{\mathrm{ic}}$ is the standard deviation of the degree of the nodes in the cluster c_i_

$$P_{i}=1-\sum_{c=1}^{M}{\left[\frac{k_{\mathrm{ic}}}{k_{i}}\right]}^{2}$$

where *k*_ic_ the number of links of node i to nodes in the cluster c, k_i_ is the total degree of node I, and M is the total number of modules in the network.

According to Guimerà and Amaral (2005) the genes were assigned to one of the following roles: ultra-peripheral nodes, peripheral, non-hub connector, non-hub kinless, provincial hubs, connector hubs, kinless hubs. These seven different roles are heuristically defined, using their localization in the different regions of the z–P parameter space (see **Supplementary Figure S1**). Nodes with z > 2.5 are classified as module hubs and nodes with z < 2.5 as non-hubs. Both hub and non-hub nodes are then further characterized by using their participation coefficient. Non-hub nodes can be divided into four different roles: ultra-peripheral nodes; that is, nodes with all their links within their module (*P* ≤ 0.05); peripheral nodes; that is, nodes with most links within their module (0.05 < *P* ≤ 0.62); non-hub connector nodes; that is, nodes with many links to other modules (0.62 < *P* ≤ 0.80); and non-hub kinless nodes; that is, nodes with links homogeneously distributed among all modules (*P* > 0.80). Similarly, hub nodes are assigned to: provincial hubs; that is, hub nodes with the vast majority of links within their module (*P* ≤ 0.30); connector hubs; that is, hubs with many links to most of the other modules (0.30 < *P* ≤ 0.75); and kinless hubs; that is, hubs with links homogeneously distributed among all modules (*P* > 0.75).

### The Analysis of Genomic Features

We used the data on germline variants detected across 60,706 exomes from the Exome Aggregation Consortium, version 0.3.1(Lek et al., 2016). To evaluate the tolerance of different sets of genes to variants in the human germline, we downloaded the data of Functional Gene Constraint^5^.

Specifically, for each human protein coding gene, we obtained the pLI (defined as the probability of being loss-of-function intolerant, including both heterozygous and homozygous LoF variants), and the pNull (defined as the probability of being tolerant to both heterozygous and homozygous LoF variants).

### Gene Expression Data in Healthy Tissues/Organs Across Individuals

We used gene expression data from GTEX (version 7.0) to analyze the pattern of expression of the different sets of genes. For GTEX tissues, we mapped the ENSEMBL gene identifiers to NCBI Gene identifiers, and kept the genes with TPM > = 1. We used the information for 53 tissues in GTEX, which represent all tissues covered except Cells.EBV.transformed.lymphocytes and Cells.Transformed.fibroblasts.

### Statistical Analysis

To compute the deviation of the value of each network feature for each set of genes, we randomly sampled 10,000 sets of genes from the network of the same size of the set under analysis in each case. Then, we computed the mean value of each sampled feature (degree, betweenness, clustering coefficient, participation coefficient, and within-module degree) for each of the 10,000 randomly sampled gene sets. From this distribution of means a *z*-score was estimated for every gene set for every feature. The same was done to compute the deviation of the value of the genomic features and of the expression of each gene set from their expected distribution, but genes were sampled from the entire list of human protein coding genes in this case. The statistical analysis were carried out using R, version 3.4.0 (R Core Team, 2017) and the network analysis were performed using the iGraph Library, version igraph_1.1.2 (Csardi and Nepusz, 2006). Additionally, the following packages were employed: UpSetR_1.3.3 (Conway et al., 2017), to produce **Figure 3**, showing the intersects between the different protein sets evaluated in the paper, and clusterProfiler_3.6.0 (Yu et al., 2012) to compare the pathways in which are involved the proteins in the class “enzyme” in the TARGET set and in the METAB set.

## Results

### More Than Half of the Proteins That Are Targets of Drugs, or Involved in Drug Metabolism Are Associated to Side Effects

We compiled three sets of drug-associated proteins as detailed in Methods (**Figure 2**). The first comprised 1,934 proteins that are well-established drug targets (TARGET set); the second comprised 470 proteins involved in drug transport and metabolism (METAB set); the third was composed of 4,160 proteins associated to Adverse Drug Reactions, or ADRs (TOXPROT set). Twenty-five percent of the proteins in the TOXPROT set are also targets of drugs (TOXPROT-TARGET, TT set). More than half of drug target proteins and of proteins involved in drug metabolism are associated to side effects (**Figure 3**).

### The Distribution of Cartographic Roles Is Preserved Across Organ-Specific Interactomes

For this study, we assembled two different global human interactomes and several organ-specific interactomes from two different resources, INBIOMAP (Li et al., 2017) and HIPPIE (Alanis-Lobato et al., 2017). We focused on brain, heart, kidney, and liver due to the relevance of these organs in drug toxicity. Throughout the paper, we illustrate the results obtained with the INBIOMAP interactomes, but all analyses were replicated in the HIPPIE-based interactomes.

The INBIOMAP global interactome is composed of 12,967 nodes and 107,787 edges. The number of proteins in organ-specific interactomes varies between 8,800 and 9,800 nodes, and the final networks contain around 80% of the interactions of the global interactome (**Supplementary Table S1**).

To uncover the modular organization of the global human interactome and the organ-specific interactomes, we employed the Infomap procedure (Rosvall and Bergstrom, 2008), one of the best performing network community recognition methodologies, which has produced biologically relevant partitions of the human interactome (Berenstein et al., 2015). After partitioning the interactome into modules, we characterized the meso-scale connectivity features for each protein in the network using the within-module degree (z) and the participation coefficient (P) parameters (Guimerà and Amaral, 2005). The z parameter standardizes the degree of a node in relation with the degree of nodes that belong to the same cluster, and the P parameter quantifies the fraction of links that a given node projects to other clusters. We further categorized each network node according to the universal cartographic role classification scheme proposed by Guimerà and Amaral (2005): ultra-peripheral, peripheral, non-hub connector, non-hub kinless, provincial hubs, connector hubs and kinless hubs (**Supplementary Figure S2**). Thus, focusing on how individual nodes are positioned in the modular (meso-scale) structure of the network, we can identify proteins that play different functions, such as those only connected to proteins within their modules, and those proteins that serve as bridges between different modules.

The cartographic analysis of the global human interactome and four organ-specific interactomes (brain, heart, kidney and liver) is shown in **Supplementary Figures S1**, **S2**, respectively, and summarized in **Table 1** and **Supplementary Table S2**. Most of the proteins in the global network have roles with within-module degree smaller than 2.5, that is kinless (14.7%), connector (28.4%), peripheral (27.5%) and ultra-peripheral (26.6%). Nodes with hub roles account for 2.8% of the network. The nodes with the higher z, also have high P, resulting in their classification as connector hub or kinless hub nodes. This distribution of genes across cartographic roles is preserved in the organ-specific networks (**Table 1**). In other words, the proportion of nodes with different roles in the network does not change substantially when we take into account only the genes expressed in each tissue to construct the networks, although there is a small decrease in the percentage of nodes in the ultra-peripheral role in organ-specific networks. A similar behavior is observed for the HIPPIE global interactome, and its organ-specific interactomes (**Supplementary Table S2**). Taken together, these findings point to a conserved network structure and connectivity patterns at the meso-scale level in the interactome across tissues.

**Table 1:** Cartographic partition of the nodes in the INBIOMAP interactomes (the global interactome, and the four organ-specific PINs).

Cartographic role	Global	Brain	Heart	Kidney	Liver
Provincial hub	9 (0.07%)	5 (0.05%)	6 (0.06%)	8 (0.08%)	6 (0.07%)
Connector hub	119 (0.92%)	68 (0.69%)	72 (0.78%)	67 (0.69%)	73 (0.82%)
Kinless hub	236 (1.82%)	216 (2.2%)	196 (2.12%)	202 (2.07%)	187 (2.1%)
Kinless	1903 (14.68%)	1718 (17.52%)	1560 (16.88%)	1603 (16.43%)	1524 (17.13%)
Connector	3686 (28.43%)	2891 (29.48%)	2808 (30.38%)	2911 (29.84%)	2692 (30.26%)
Peripheral	3570 (27.53%)	2604 (26.56%)	2528 (27.35%)	2660 (27.27%)	2403 (27.01%)
Ultra-peripheral	3444 (26.56%)	2303 (23.49%)	2074 (22.44%)	2303 (23.61%)	2011 (22.61%)

*The number of nodes in each cartographic role for each network is shown, with its percentage between parentheses.*

### Targets That Mediate Side Effects and Side Effect Proteins Are Important for Connecting Different Modules in the Network

Next, we studied the multi-scale network properties of the sets of genes relevant for drug response within the context of the global and the organ-specific interactomes. The coverage for the different gene sets in the interactomes varies between 70 and 90% in the INBIOMAP global interactome (**Supplementary Table S3**). Eighty percent of the TARGET and TOXPROT sets are present in the global networks, while METAB proteins coverage is the lowest (around 70%). The coverage in the organ-specific networks ranges between 50 and 60% depending on the protein set and the tissue. Similar coverage is observed for the HIPPIE-based interactomes (**Supplementary Table S3**).

The analysis of the proteins belonging to each set according to their cartographic role showed that TARGET proteins are significantly enriched for nodes that play kinless and kinless hub roles in the network (**Table 2**). The enrichment of TOXPROT proteins is more apparent for nodes of the network that play kinless, kinless hub and marginally connector roles. As a matter of fact, the overrepresentation of targets in the kinless and kinless hub nodes is almost completely explained by the subset of TT amongst them (targets that are associated to side effects). METAB proteins are not particularly enriched in any role in the network. The results are similar in organ-specific interactomes (**Supplementary Figure S3**) and for networks derived from HIPPIE, except for the case of METAB proteins, which play peripheral roles in the global and the liver HIPPIE PINs (**Supplementary Figure S3**). The distribution of the gene sets across the seven cartographic roles is shown in **Supplementary Table S4**.

**Table 2:** Enrichment analysis of the cartographic roles of each set of genes in the INBIOMAP global interactome.

Cartographic role	TARGET	TT	OT	METAB	TOXPROT	OTP
Kinless hub	3.4 (1.2*e*–14)	4.2 (8.7*e*–15)	1.7 (0.09)	0.66 (1.0)	2.6 (1.1*e*–11)	1.3 (1.1*e*–01)
Connector hub	1.4 (2.2*e*–01)	2.2 (1.9*e*–02)	0.46 (1.0)	0.32 (1.0)	1.4 (1.0*e*–01)	1 (7.8*e*–01)
Provincial hub	3.5 (2.0*e*–01)	1.6 (7.9*e*–01)	5.1 (0.19)	0 (1.0)	1.4 (7.8*e*–01)	1.2 (8.5*e*–01)
Kinless	1.7 (1.5*e*–13)	1.9 (1.7*e*–12)	1.3 (0.02)	1 (0.78)	2 (3.5*e*–40)	1.8 (3.8*e*–23)
Connector	1.1 (1.0*e*–01)	1.1 (1.1*e*–01)	1.1 (0.54)	0.86 (1.0)	1.2 (1.7*e*–03)	1.1 (1.6*e*–02)
Peripheral	0.85 (1.0)	0.74 (1.0)	1 (0.6736)	0.93 (1.0)	0.8 (1.0)	0.86 (1.0)
Ultra-peripheral	0.55 (1.0)	0.49 (1.0)	0.69 (1.0)	1.3 (0.06)	0.55 (1.0)	0.62 (1.0)

*The fold enrichment of the Fisher’s exact test is shown with the corresponding p-value corrected by the Benjamini and Hochberg method between parentheses. The cartographic partition of the different gene sets in the INBIOMAP and HIPPIE interactomes is provided in **Supplementary Table S3**.*

**FIGURE 4:**
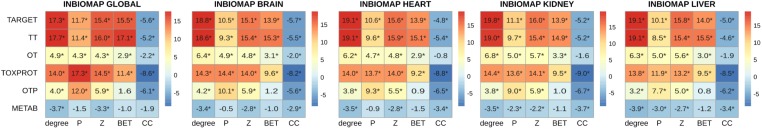
Multi-scale network features of the gene sets (INBIOMAP interactomes). We plot the *z*-score of the network features (degree, P: participation coefficient, Z: within-module degree, BET: betweenness, CC: clustering coefficient) resulting from 10,000 randomizations. The asterisks indicate that the *z*-score is statically significant (*p*-value < 0.05). TARGET: drug targets, METAB: proteins that are involved in the drug metabolism, absorption, distribution, metabolism, and excretion. TOXPROT: proteins associated to side effects. TT: genes in common between drug targets and toxicity genes. OT: only TARGET proteins. OTP: only TOXPROT proteins.

A more detailed analysis of other network properties of the sets of genes shows that TARGET, TOXPROT, and TT sets tend to have a significantly higher degree, participation coefficient, within-module degree, and betweenness than the other genes in the network (**Figure 4**). They also have a lower clustering coefficient. We note, however, that most of the effect observed for the TARGET and TOXPROT sets is explained by their shared TT subset. On the other hand, METAB proteins have significantly lower degree, and within module degree than expected and significantly lower participation coefficient in most organ-specific interactomes (**Figure 4**). METAB proteins are more specialized, thus it would make sense that they play less central roles in the network, with less interaction partners in the interactome. Similar results are obtained for HIPPIE interactomes (**Supplementary Figure S4**).

### Drug Targets, and Toxicity Proteins Are Highly Sensitive to Loss of Function Mutations, While Proteins Involved in Drug Metabolism Are Tolerant

Next, we analyzed the tolerance of drug related proteins to LoF variants using exome sequence data from 60K “healthy” subjects provided by the ExAC consortium (Lek et al., 2016). We employed two gene constraint metrics developed by the ExAC team: pLI and pNull. pLI is the probability of a gene to be intolerant to heterozygous LoF mutations (LoF variants are nonsense and essential splice site variants). It separates genes into LoF intolerant (pLI ≥ 0.9) or LoF tolerant (pLI ≤ 0.1). On the other hand, pNull is the probability of a gene to be tolerant to both heterozigous and homozigous LoF variation.

We found that METAB genes have significantly lower pLI than the other genes in the genome (**Table 3**). In other words, METAB genes are more tolerant to LoF variation than the average human genes. On the other hand, TARGET and TOXPROT genes have significantly greater pLI value than average human genes. Since genes intolerant to LoF variation are likely to be dosage sensitive (Lek et al., 2016), TARGET and TOXPROT sets might contain haploinsufficient genes. The results for the pNull are consistent with those of the pLI, but with the opposite meaning: genes with high pNull are tolerant to LoF variation.

**Table 3:** Genomic features of the sets of genes.

Gene set	pLI			pNull		
	pLI	*z*-score	*p*-value	pNull	*z*-score	*p*-value
TARGET	0.380	8.87	7.31E-19	0.167	−5.829	5.58E-09
TOXPROT	0.365	10.9	1.15E-27	0.146	−13.604	3.79E-42
METAB	0.214	−4.92	8.65E-07	0.260	3.843	1.22E-04
TT	0.399	7.84	4.51E-15	0.153	−5.607	2.06E-08
OT	0.358	4.15	3.32E-05	0.183	−2.239	0.0251559
OTP	0.354	7.45	9.33E-14	0.143	−12.108	9.58E-34

*We show the value of the feature, the z-score resulting from 10,000 randomizations, and its associated two-sided p-value.*

**FIGURE 5:**
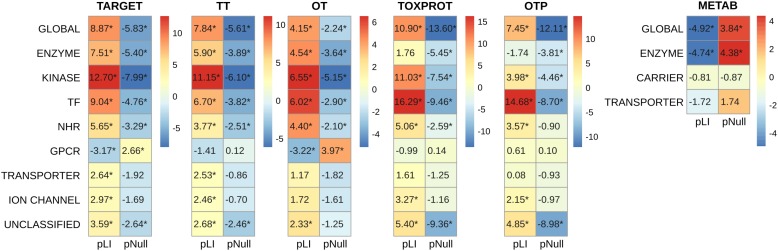
Genomic features of the sets of genes, and of the protein classes. We plot the *z*-score resulting from 10,000 randomizations. The asterisks indicate that the *z*-score is statically significant (*p*-value < 0.05). TARGET: drug targets, METAB: proteins that are involved in the drug metabolism, absorption, distribution, metabolism, and excretion. TOXPROT: proteins associated to side effects. TT: genes in common between drug targets and toxicity genes. OT: only TARGET proteins. OTP: only TOXPROT proteins. See **Figure 1** for more details. It is worth noting that while 90% genes from the total of 1,934 TARGET genes are classified in the 7 target classes, 40% of the TOXPROT set belongs to other protein classes (Unclassified set).

In order to explore in more detail the features of different classes of TARGET proteins, we classified them using categories from the drug target ontology (Lin et al., 2017) (**Figure 5**). We used similar categories to classify the TOXPROT set (for more details see Methods section), and we classified METAB genes into carriers, enzymes, and transporters using the information from DrugBank. We found that among METAB genes, enzymes display the lowest pLI and highest pNull, while carriers and transporters are not significantly different than expected in terms of pLI (**Figure 5**). In the TARGET set, kinases are the most intolerant subset to LoF variation, with a mean value more than 12 SD greater than the expected mean pLI value, followed by transcription factors (*z*-score = 9.04) and TARGET enzymes (*z*-score = 7.51). It is worth noting that the enzymes within the TARGET set are related to signaling pathways, and core cellular metabolic processes, while the enzymes in the METAB set are proteins mainly participating in the metabolism of xenobiotics (**Supplementary Figure S5**). The remaining groups of TARGET genes are also intolerant to LoF but to a lesser extent, with the exception of GPCRs, that are more tolerant to LoF variation than expected (**Figure 5**). Again, the results for pNull are consistent with those of pLI, except for the case of ion channels, which are marginally intolerant to LoF variation, but do not show differences with the rest of the genes with respect to pNull.

Within the TOXPROT set, transcription factors exhibited the highest intolerance to LoF variants, followed by kinases. Nevertheless, the enzymes were not different than the rest of the genes, but they do show a lower pNull, indicating that they are less tolerant to LoF than the background.

### Proteins Associated to Side Effects Are Highly Expressed Across Tissues

**FIGURE 6:**
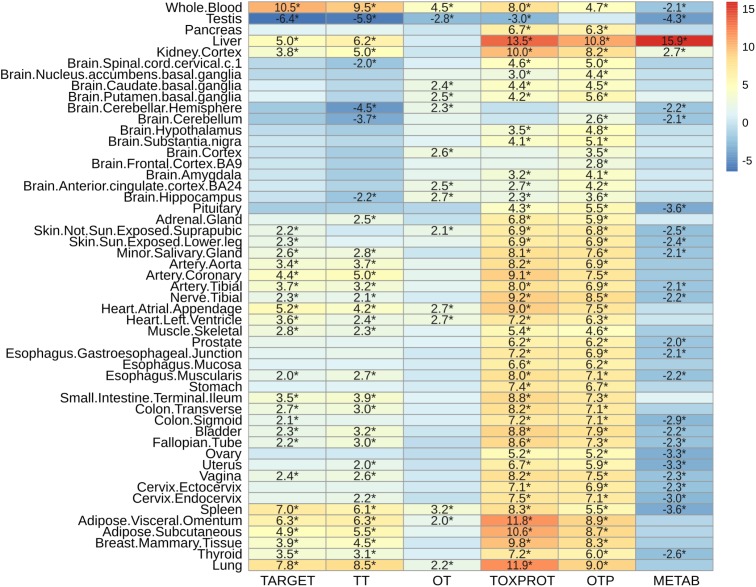
Characterization of the expression level by tissue of the sets of genes. We plot the *z*-score resulting from 10,000 randomizations. The asterisks indicate that the *z*-score is statically significant (*p*-value < 0.05). TARGET: drug targets, METAB: proteins that are involved in the drug metabolism, absorption, distribution, metabolism, and excretion. TOXPROT: proteins associated to side effects. TT: genes in common between drug targets and toxicity genes. OT: only TARGET proteins. OTP: only TOXPROT proteins.

Next, we characterized the expression patterns of each gene set across normal human tissues, using GTEx data (**Figure 6**). TOXPROT genes are more expressed than other genes in the genome across all tissues, with the exception of some areas of the brain (that do not show statistically significant differences with the other genes), and the testis (where they show a lower level of expression than other genes of the genome). TARGETs also tend to be more highly expressed than other genes of the genome across most tissues. The tissues with the most significantly higher expression are blood, lung, spleen, liver, adipose tissue, and heart. Drug targets are not significantly over or under expressed in any brain area. A closer look at this set shows that TT tend to behave like the TARGET set, with the exception of few brain areas, such as cerebellum and cerebellar hemisphere. On the other hand, OTs are not expressed at higher levels than other genes of the genome, with very few exceptions, which exhibit marginal significance. Probably, drug targets that are expressed in more tissues throughout the body, at higher levels than the rest of the proteins are more likely to elicit side effects. The broader the expression of the target, the higher is the risk of adverse reactions when the drug is administered systemically (Gashaw et al., 2011).

As expected, metabolic enzymes exhibited most significantly higher expression in liver, and to some extent in kidney, but they tend to show significantly lower expression in most tissues. Interestingly, the levels of expression of all sets of proteins (except OTP) in testis are significantly lower than the other genes in the genome.

## Discussion

There is a pressing need to identify “good” targets for safer drug development, patient treatment, and better management of drug toxicity. In this contribution, we propose that leveraging large scale genomic, transcriptomic, and interactomic data can support this goal. We show that drug targets, targets associated to side effects, and proteins associated to side effect display higher level of centrality measures (degree, betweenness, z, P and lower clustering coefficient) in the protein interaction network, indicating that they occupy central positions in the network. This centrality is evidenced not only at the local network level (evidenced by the degree and clustering coefficient), but more importantly, they are key nodes for connecting different modules in the network. On the other side, proteins participating in drug metabolism are characterized by lower degree and within-module degree, and their role is confined to their own modules in the network.

**FIGURE 7:**
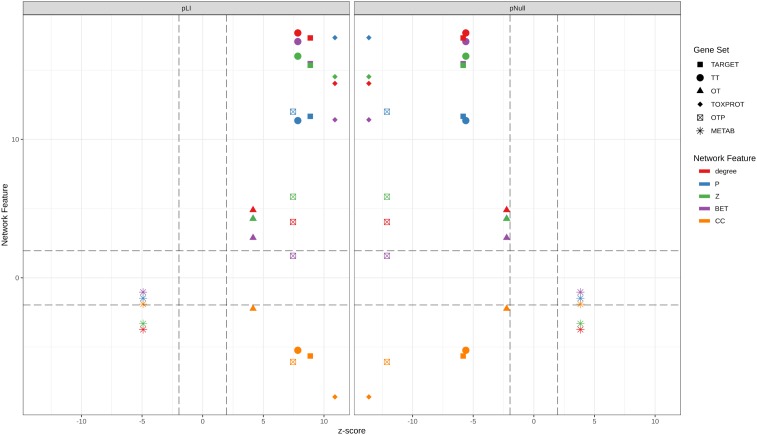
Relationship between the network features (degree, betweenness, clustering coefficient, participation coefficient, and within-module degree) and the tolerance to LoF variation (pLI and pNull) of the gene sets in the INBIOMAP global interactome. We plot the *z*-scores of the genomic and the network features resulting from 10,000 randomizations. TARGET: drug targets, METAB: proteins that are involved in the drug metabolism, absorption, distribution, metabolism, and excretion. TOXPROT: proteins associated to side effects. TT: genes in common between drug targets and toxicity genes. OT: only TARGET proteins. OTP: only TOXPROT proteins.

We then assessed how the observed differences in these network properties are related to the tolerance of each set of genes to LoF variation. We observed that genes which play more central roles in the network exhibit significantly lower tolerance to LoF variants, indicating that they are under stronger purifying selection. These results are in agreement with observations across genes related to different disease classes (Piñero et al., 2016a). On detail, we found that disease genes that play central roles in the network, such as cancer genes and genes associated to autosomal dominant diseases show less tolerance to likely deleterious variants than genes associated to autosomal recessive diseases, which play peripheral roles in the network (Piñero et al., 2016a). **Figure 7** shows the separation between METAB genes, and TARGET and TOXPROT in terms of the gene constraint metrics (pLI and pNull) and the multi-scale network features.

We found that TT genes are more central in the network, (indicated by their higher z, degree and P, and lower clustering coefficient), than OT genes. In particular, the observed higher P indicates that these proteins play an important role in connecting different modules within the network, suggesting that they are pleiotropic and participate in diverse biological processes, which could explain why they are mediators of both, therapeutic and side effects of drugs. These results are in line with those of Perez-Lopez et al. (2015) who showed that drug targets that mediate side effects are better spreaders of perturbations in a human global interactome, than targets of drugs having no reported side effects, and non-target proteins. Our results also support those of Kotlyar et al. (2012) who showed that drug targets and drug-regulated genes have higher degree and betweenness, and lower clustering coefficients.

Drug targets and drug targets that cause side effects are significantly LoF intolerant, while METAB proteins are relatively tolerant to LoF variants and to homozygous LoF variants (**Figure 7**). These results agree with those of a recent study (Wright et al., 2018) that found high-confidence LoF variants in more than half of the pharmacogenes under analysis. The relatively high tolerance of METAB proteins may be at least partially explained by their degree of paralogy (Pan et al., 2016), and overlapping substrate specificity across these enzymes (Zhou, 2008). In detail, there are 32 cytochromes in the METAB dataset, and their drug specificity ranges from 1 to over 600 drugs. They are all characterized by their relatively high tolerance to LoF mutations (e.g., low pLI values and high pNull values). An example of redundancy in these enzymes is CYP3A5 (pLI = 5.2 e-11). It has been reported that CYP3A5 deficiency occurs in approximately 75% of white persons and 50% of African descent populations because of a single nucleotide polymorphism (CYP3A5^∗^3, 6986A > G) within intron 3 that introduces a premature stop codon and truncation of the protein (Kuehl et al., 2001). Because many drugs metabolized by CYP3A5 are also substrates of CYP3A4, truncating mutations in either of these proteins might produce no visible phenotype.

The intolerance to LoF variation observed in the TARGET set is mainly driven by TT genes, as shown by the smaller z-scores of pLI, and pNull of OT genes (**Figure 7**). GPCRs behave differently than the other TARGET classes. They possess lower pLI and higher pNull values than the rest of the genes. A closer look at this set of proteins shows that GPCRs that do not directly mediate side effects (OT set, 89 genes with ExAC data) are responsible for this trend, since GPCRs in the TT set (123 genes with ExAC data) have no significantly different pLI values than the rest of the genes. GPCRs do not seem to play central roles in the network at the global and meso-scale level (although they display low clustering coefficient, see **Supplementary Figure S6**), which suggests that they are not under strong negative selection and therefore would be more tolerant to functional variants. A recent study of the pharmacogenomics of 108 GPCRs targeted by FDA approved drugs (Hauser et al., 2018) showed that GPCRs have, on average, LoF mutations in 9 different positions per receptor, and at least 1 LoF variant has been observed in each of the GPCRs under study. The mechanisms that might explain the compatibility of these drastic genomic alterations with normal phenotypes could be heterozygosity, epistasis, and allele-specific expression. Nevertheless, it is also possible that some of the receptors with low pLI have functional redundancy.

The fact that drug targets that mediate side effects tend to be more intolerant to LoF variation is in line with the finding that the inter individual genomic variability of drug targets is a strong predictor of the withdrawal of drugs (Lee et al., 2016). This study, using several metrics to estimate the deleteriousness of variants in 2,504 publicly available genomes from the 1000 Genomes Project, found a high person-to-person variability of deleterious variants among drug-related genes. They also designed a genomic deleteriousness score that they found to be significantly lower for withdrawn drugs, and US FDA pharmacogenomic biomarkers than for other drug-related proteins.

Finally, we have characterized the expression of drug related genes across healthy human tissue, showing differences in the pattern of expression among the different gene sets. To the best of our knowledge, this is the first study that performs such analysis.

Our results show that there is a relationship between the role in the cellular network of genes involved in different drug effects and their tolerance to LoF variation. We have uncovered a scenario in which proteins that mediate side effects are more central, tend to be more intolerant to LoF mutations, and are highly expressed in most of the human tissues. The subset of drug targets that mediate drug adverse reactions occupy more central positions in the network –not only because they have a high degree, but because they connect different network modules–, and they also exhibit higher sensitivity to LoF variants. In contrast, drug targets that do not mediate side effects do not exhibit any significant pattern of network centrality, and appear to be under weaker negative selection. The case of ADME proteins is particular, because they are less central, tolerate LoF mutations, and show a very specific tissue expression pattern. The integrated analysis of different omics data reveals distinct features of proteins associated to drug response, which is relevant in the context of drug development and pharmacogenomics.

## Author Contributions

JP and LF conceived and designed the experiments. JP performed the experiments. JP, AG-P, EG, and LF analyzed the data. JP, AG-P, EG, LF, JA-P, FS, and BO reviewed and discussed the results. JP, LF, and AG-P wrote the paper. All the authors reviewed the final version of the manuscript.

## Conflict of Interest Statement

The authors declare that the research was conducted in the absence of any commercial or financial relationships that could be construed as a potential conflict of interest.

## Footnotes

1 https://www.fda.gov/Drugs/ScienceResearch/ucm572698.htm

2 ftp://ftp.pantherdb.org/sequence_classifications/current_release/PANTHER_Sequence_Classification_files/PTHR13.1_human

3 https://www.intomics.com/inbio/map/#downloads

4 http://cbdm-01.zdv.uni-mainz.de/~mschaefer/hippie/hippie_current.txt

5 ftp://ftp.broadinstitute.org/pub/ExAC_release/release0.3.1/functional_gene_constraint/README_fordist_cleaned_exac_r03_z_data_pLI_2016_01_13.txt

## Abbreviations

LoF: loss-of-function variants, including variants affecting splice sites, or stop codons
METAB: proteins that are involved in the drug metabolism, absorption, distribution, metabolism, and excretion
OT: drug targets that do not mediate side effects
OTP: proteins associated to side effects that are not drug targets
TARGET: drug targets
TOXPROT: proteins associated to side effects
TT: drug targets that mediate side effects.
