# Supplementary material for: Network, Transcriptomic and Genomic Features Differentiate Genes Relevant for Drug Response
Source: Front Genet. 2018 Sep 25;9:412. doi: 10.3389/fgene.2018.00412 (PMC6168038; doi:10.3389/fgene.2018.00412)
Supplement: Supplementary file 1 [file Table_1.docx]

Supplementary Material

Network, Transcriptomic and Genomic Features Differentiate Genes Relevant for Drug Response

Janet Piñero, Abel Gonzalez-Perez, Emre Guney, Joaquim Aguirre-Plans, Ferran Sanz, Baldo Oliva, Laura I. Furlong*

*** Correspondence:** Laura I. Furlong: laura.furlong@upf.edu

# Supplementary Data

# Supplementary Figures and Tables

Supplementary Table 1: Number of nodes and edges of the interactomes used in this study.

| **Source** | **Interactome** | **Nodes** | **Edges** |
| --- | --- | --- | --- |
| INBIOMAP | Global | 1,2967 | 107,787 |
|  | Brain | 9,805 | 76,209 |
|  | Heart | 9,244 | 74,121 |
|  | Kidney | 9,754 | 77,825 |
|  | Liver | 8,896 | 72,329 |
| HIPPIE | Global | 13,007 | 90,792 |
|  | Brain | 10,089 | 69,293 |
|  | Heart | 9,563 | 66,326 |
|  | Kidney | 10,014 | 69,595 |
|  | Liver | 9,147 | 63,986 |

Supplementary Table 2: Cartography of the HIPPIE interactomes (the global interactome, and the four tissue-specific interactomes). The number of nodes (and their percentage) in each cartographic role and interactome is shown.

| **Cartographic Role** | **Global** | **Brain** | **Heart** | **Kidney** | **Liver** |
| --- | --- | --- | --- | --- | --- |
| provincial hub | 27 (0.21%) | 9 (0.09%) | 8 (0.08%) | 9 (0.09%) | 12 (0.13%) |
| connector hub | 94 (0.72%) | 82 (0.81%) | 83 (0.87%) | 80 (0.8%) | 77 (0.84%) |
| kinless hub | 263 (2.02%) | 195 (1.93%) | 177 (1.85%) | 191 (1.91%) | 166 (1.81%) |
| kinless | 3,116 (23.96%) | 2,478 (24.56%) | 2,256 (23.59%) | 2,429 (24.26%) | 2,171 (23.73%) |
| connector | 3,544 (27.25%) | 2,786 (27.61%) | 2,783 (29.1%) | 2,843 (28.39%) | 2,648 (28.95%) |
| peripheral | 3,028 (23.28%) | 2,502 (24.8%) | 2,381 (24.9%) | 2,473 (24.7%) | 2,310 (25.25%) |
| ultra-peripheral | 2,935 (22.56%) | 2,037 (20.19%) | 1,875 (19.61%) | 1,989 (19.86%) | 1,763 (19.27%) |

Supplementary Table 3: Coverage of the different gene sets in the INBIOMAP and HIPPIE interactomes, in percentage.

| **Source** | **Set** | **GLOBAL** | **BRAIN** | **HEART** | **KIDNEY** | **LIVER** |
| --- | --- | --- | --- | --- | --- | --- |
| INBIOMAP | TARGET | 84.0 | 59.4 | 57.0 | 59.6 | 54.5 |
|  | TT | 91.2 | 62.6 | 62.1 | 64.6 | 58.7 |
|  | OT | 75.7 | 55.5 | 51.0 | 53.8 | 49.6 |
|  | TOXPROT | 83.2 | 61.7 | 59.6 | 62.5 | 57.7 |
|  | OTP | 80.6 | 61.5 | 58.8 | 61.9 | 57.4 |
|  | METAB | 70.2 | 45.1 | 40.2 | 51.7 | 50.0 |
| HIPPIE | TARGET | 80.7 | 58.4 | 56.1 | 58.7 | 53.8 |
|  | TT | 86.4 | 60.5 | 60.2 | 63.1 | 57.2 |
|  | OT | 74.0 | 55.8 | 51.3 | 53.5 | 49.8 |
|  | TOXPROT | 81.1 | 61.7 | 59.9 | 62.2 | 57.5 |
|  | OTP | 79.4 | 62.0 | 59.8 | 62.0 | 57.6 |
|  | METAB | 65.7 | 45.1 | 42.3 | 50.2 | 48.1 |

Supplementary Table 4: Cartographic roles of the different gene sets in the INBIOMAP and HIPPIE global interactomes. The number of nodes in each cartographic role and gene set is shown.

| **Source** | **Cartographic Role** | **TARGET** | **TT** | **OT** | **TOXPROT** | **OTP** | **METAB** |
| --- | --- | --- | --- | --- | --- | --- | --- |
| INBIOMAP | provincial hub | 3 | 1 | 2 | 3 | 2 | 0 |
|  | connector hub | 20 | 17 | 3 | 41 | 24 | 1 |
|  | kinless hub | 76 | 56 | 20 | 113 | 57 | 4 |
|  | kinless | 344 | 217 | 127 | 757 | 540 | 50 |
|  | connector | 492 | 288 | 204 | 1062 | 774 | 84 |
|  | peripheral | 402 | 207 | 195 | 842 | 635 | 86 |
|  | ultra-peripheral | 285 | 145 | 140 | 642 | 497 | 105 |
|  | totals | 1622 | 931 | 691 | 3460 | 2529 | 330 |
|  | coverage | 83.9 | 91.2 | 75.7 | 83.2 | 80.6 | 70.2 |
| HIPPIE | provincial hub | 5 | 2 | 3 | 5 | 3 | 1 |
|  | connector hub | 14 | 8 | 6 | 34 | 26 | 4 |
|  | kinless hub | 67 | 50 | 17 | 109 | 59 | 5 |
|  | kinless | 440 | 255 | 185 | 1001 | 746 | 60 |
|  | connector | 411 | 230 | 181 | 944 | 714 | 77 |
|  | peripheral | 347 | 185 | 162 | 680 | 495 | 63 |
|  | ultra-peripheral | 274 | 152 | 122 | 602 | 450 | 99 |
|  | totals | 1553 | 880 | 673 | 3370 | 2490 | 308 |
|  | coverage | 80.3 | 86.2 | 73.7 | 81.0 | 79.3 | 65.5 |

## Supplementary Figures


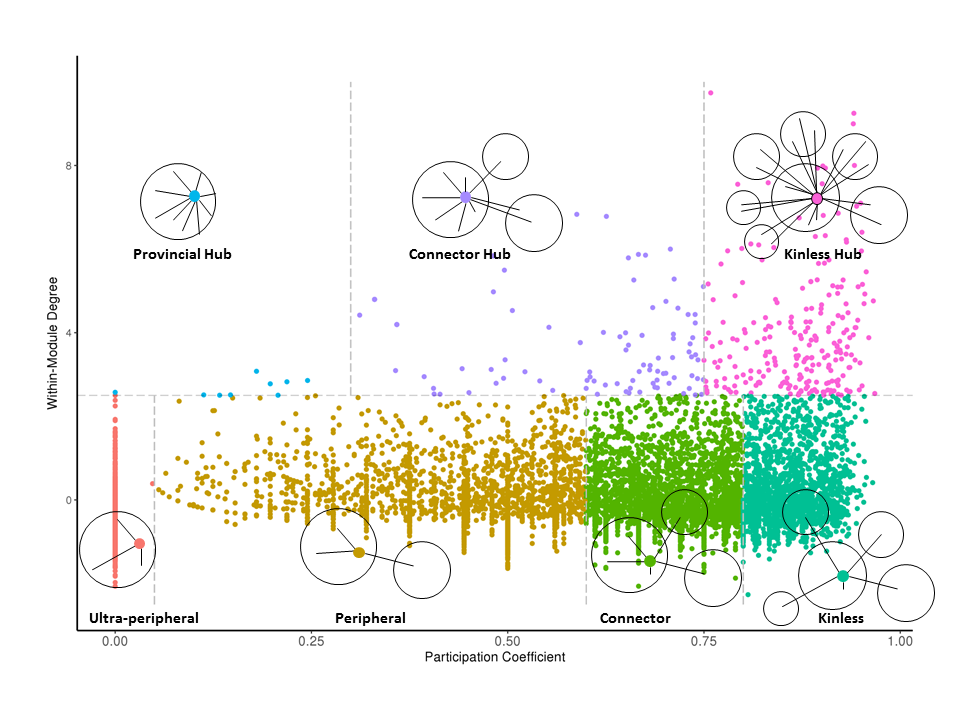


**Supplementary Figure 1:** Cartographic partition of the nodes in the INBIOMAP global interactome. The cartographic roles are represented in the z-P plane with different colors. Dashed lines in the figure delineate regions corresponding to the seven cartographic roles. We show a schematic representation of the type of connection for each role. Nodes with z > 2.5 are classified as module hubs and nodes with z < 2.5 as non-hubs. Both hub and non-hub nodes are then further characterized by using their participation coefficient. Ultra-peripheral and peripheral nodes are genes with a lower degree than the members of their cluster, that have all (ultra-peripheral nodes), and most of their links (peripheral nodes) within their module. Connector nodes have many links to other modules, while kinless nodes have their links homogeneously distributed among all modules. Finally, provincial hubs are those genes that are more connected than their neighbors in the cluster, but they have most of their connections inside their cluster. Thus, provincial hub nodes are important in maintaining the local structure of their own modules, while connector and kinless nodes are relevant for connecting different modules (and therefore different biological processes) and in maintaining the global connection of the network.


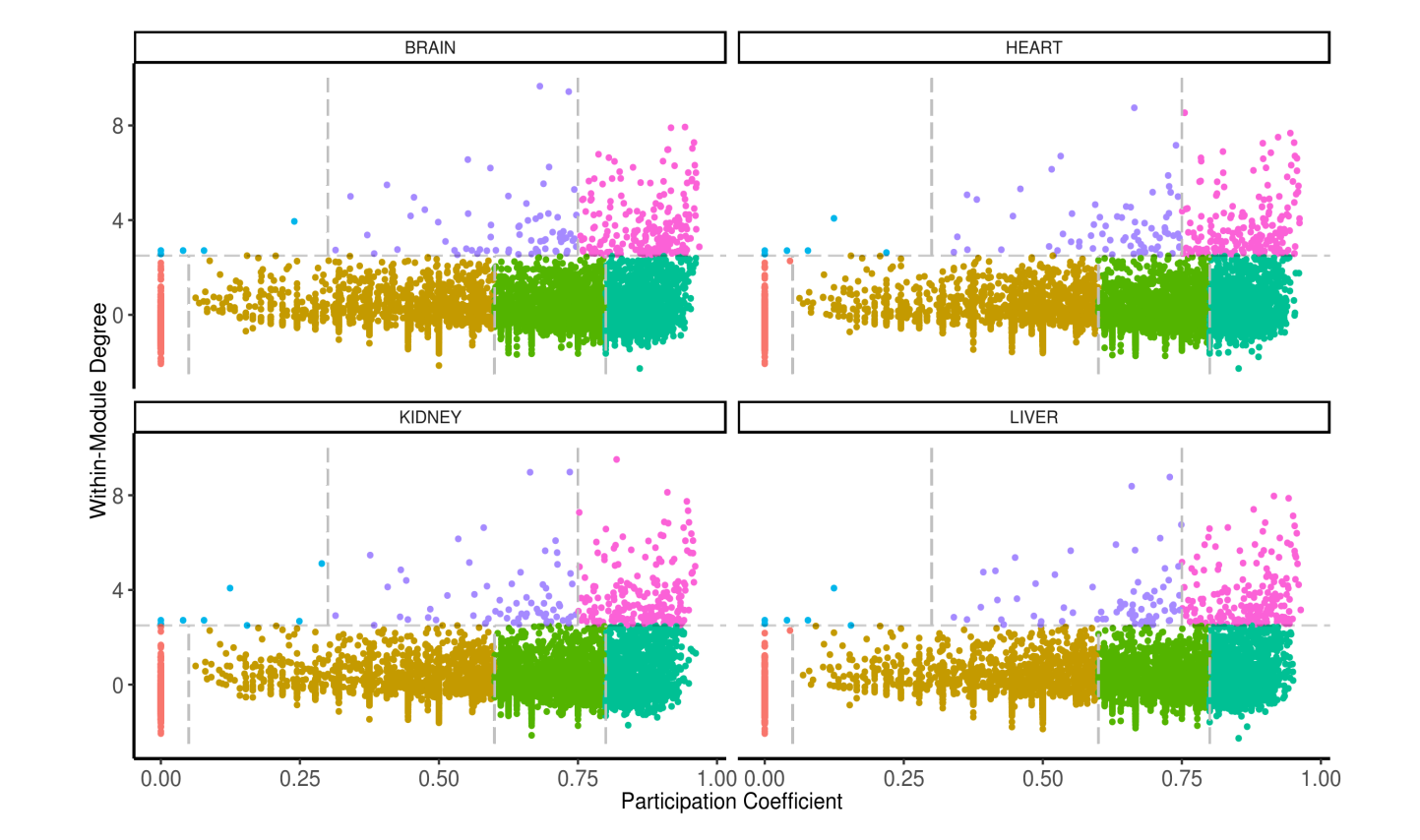


**Supplementary Figure 2:** Cartographic partition of the nodes in the INBIOMAP tissue-specific interactomes. The cartographic roles are represented in the z-P plane with different colors. Dashed lines in the figure delineate regions corresponding to the seven cartographic roles.


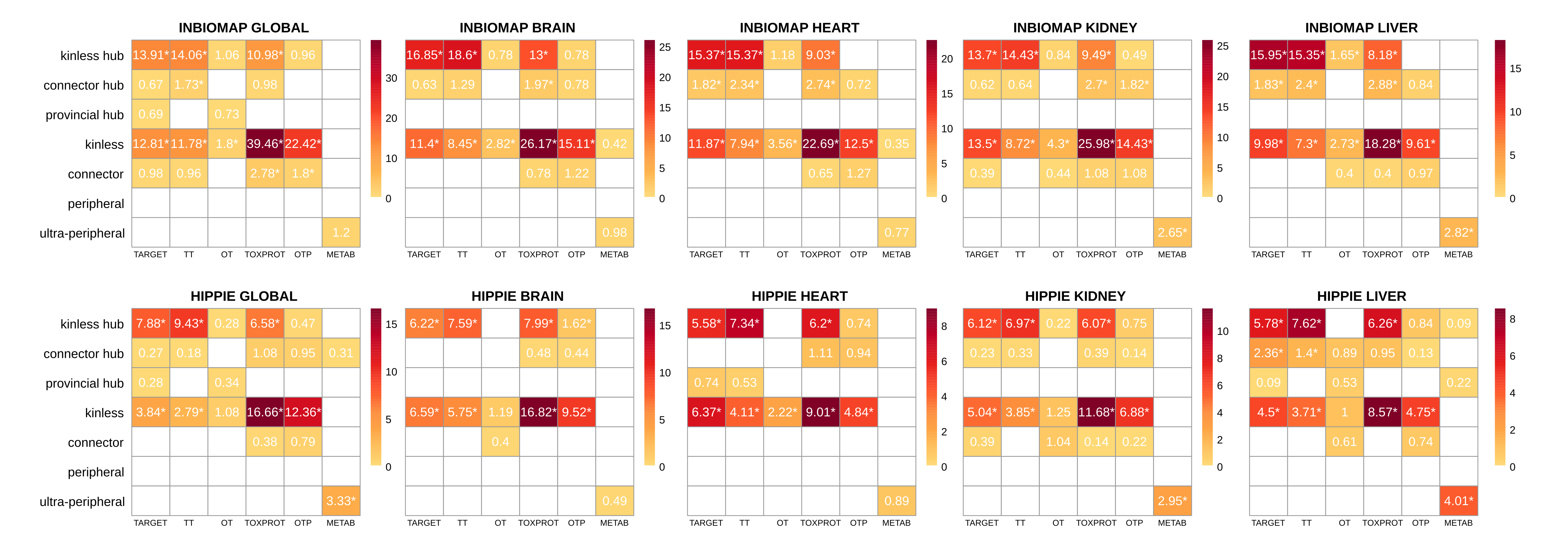


**Supplementary Figure 3:** Enrichment of the gene sets in each cartographic role in the global and tissue-specific interactomes from INBIOMAP and HIPPIE. The color scale is proportional to the p-value of the Fisher’s exact test corrected by the Benjamini & Hochberg method.

**
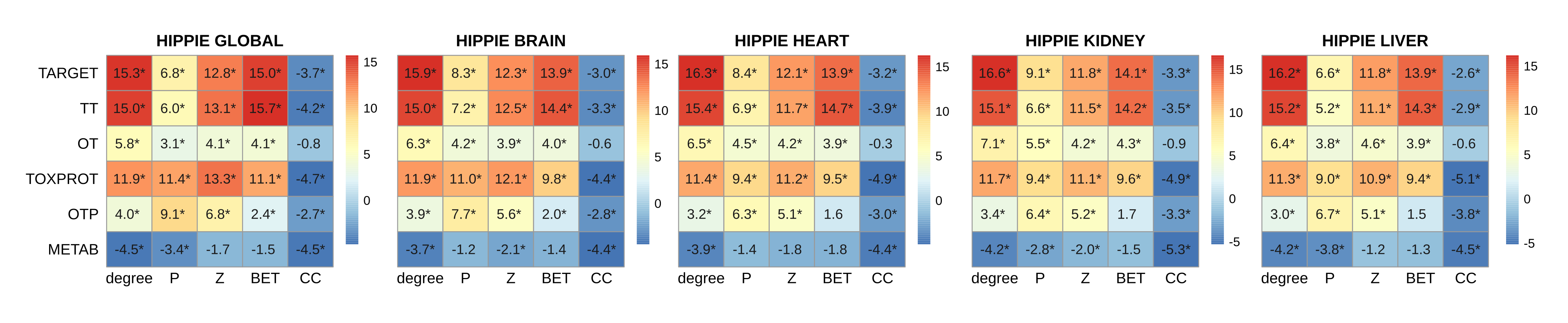
**

**Supplementary Figure 4:** Network features (degree, P: participation coefficient, Z: within-module degree, BET: betweenness, CC: clustering coefficient) of the gene sets (HIPPIE interactomes).We plot the z-score of the network features resulting from 10,000 randomizations. TARGET: drug targets, METAB: proteins that are involved in the drug metabolism, absorption, distribution, metabolism, and excretion. TOXPROT: proteins associated to side effects. TT: genes in common between drug targets and toxicity genes. OT: only TARGET proteins. OTP: only TOXPROT proteins.

**
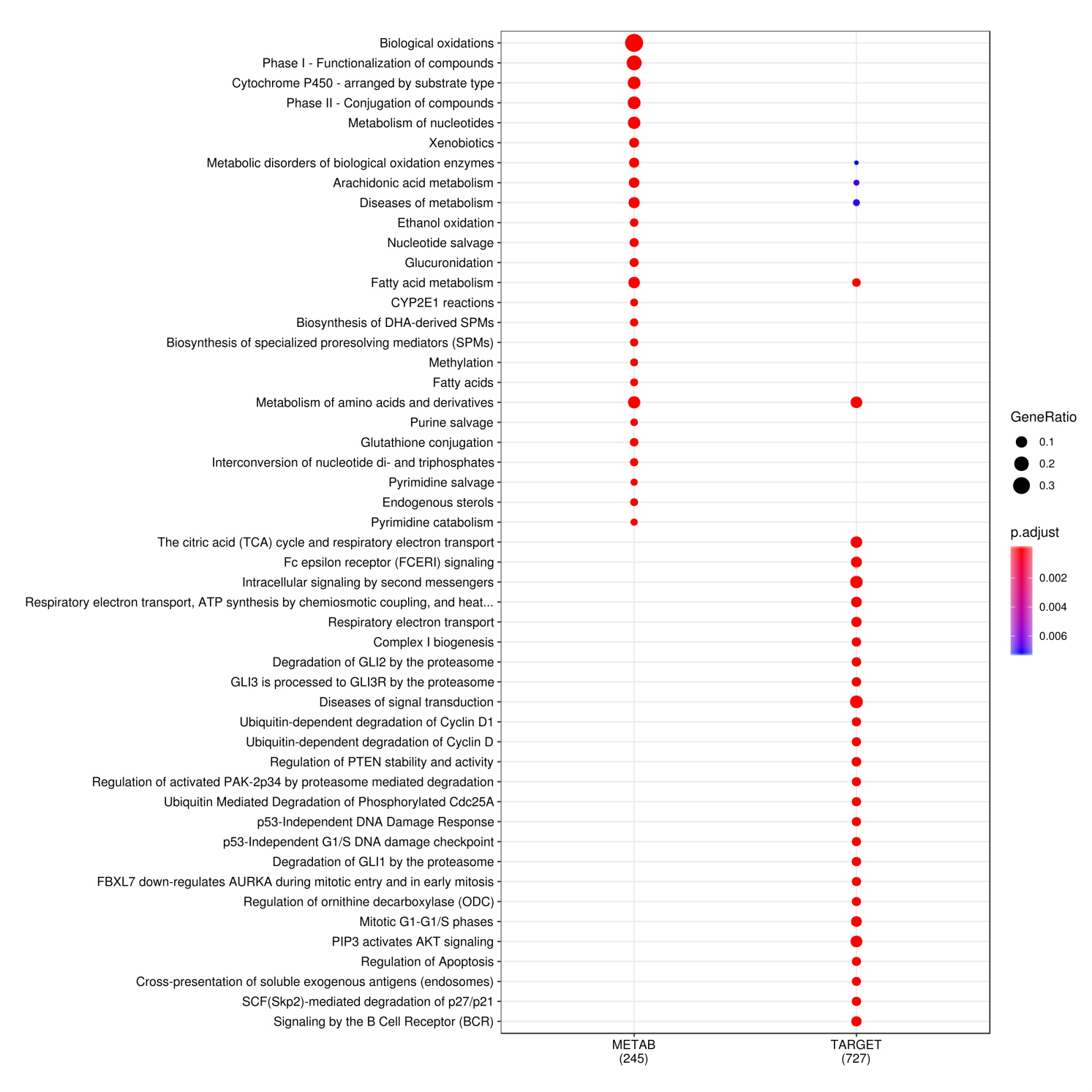
Supplementary Figure 5:** Pathway Enrichment for the subset of enzymes in the TARGET set and in the METAB set. We show the top 25 Reactome pathways (ordered by decreasing p-value). The color of the dot is proportional to the corrected p-value of the Fisher’s exact test, and the size of the dot, to the ratio of the number of genes in the set in the pathway and the total number of genes in the set that map to at least one pathway.


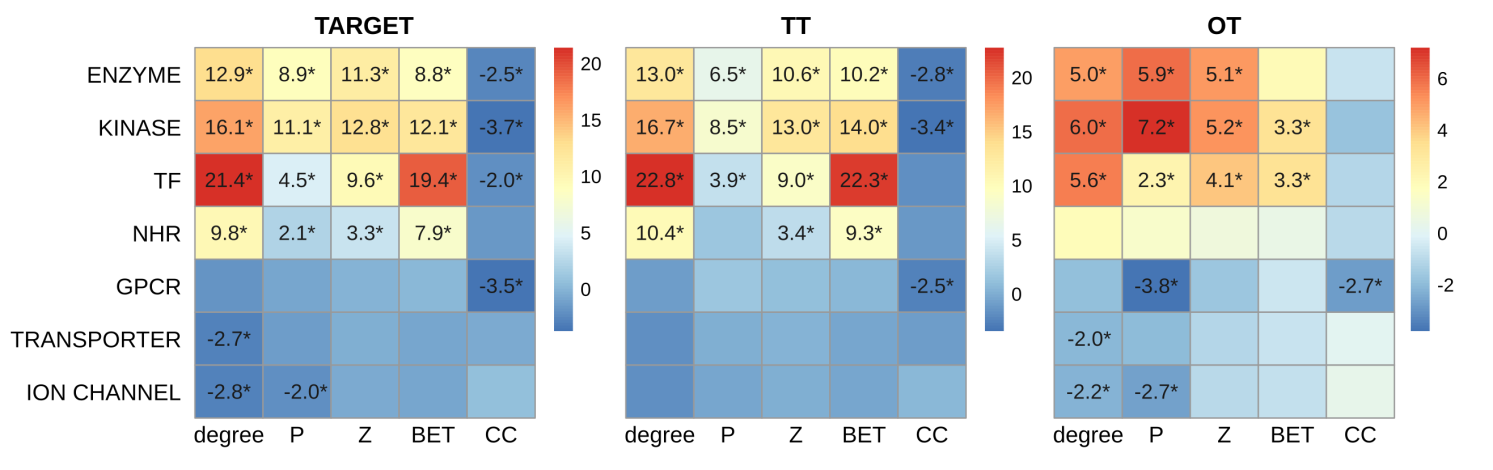


**Supplementary Figure 6:** Network features (degree, P: Participation coefficient, Z: within-module degree, BET: betweenness, CC: clustering coefficient) of the gene sets (HIPPIE interactomes) of the protein classes for the TARGET, TT, and OT sets in the global INBIOMAP interactome. We plot the z-score of the network features resulting from 10,000 randomizations. TARGET: drug targets, TT: genes in common between drug targets and toxicity genes. OT: only TARGET proteins.
